# Coffee

**Published:** 1858-06

**Authors:** 


### COFFEE.

Considering our habits of life, coffee, as a beverage for breakfast, is nutritious and healthful, and may be taken in moderation, for a lifetime, without failing of its advantageous effects. A single cup, moderately strong, never increased in strength, frequency, or quantity, is a positive good, and is far better than as much cold water at any meal, especially to invalids, or persons of a feeble digestion. Now and then, a person will be found with whom it does not agree; then such a person should omit it, at least, for a time. To obtain coffee with its delicious aroma, it should be boiled in a vessel which shall keep in all the steam, *every particle of it;* and then, the longer it is boiled, the better it is, even if for two hours. *The Old Dominion Coffee Pot*, patented by Arthur Burnham & Gilroy of Philadelphia, accomplishes this most certainly, as we personally know, and is claimed to be a saving of twenty-five per cent—that is, three pounds will go as far as four. But to make it of any use whatever, one thing is essential: the purchaser must first learn how the coffee is made, and then remain in the kitchen while the cook is directed how to make it, and seeing that the directions are literally attended to. After this is done for two or three mornings, neither mistress nor maid will have any desire to return to the old plan, of boiling all the best part away, and allowing its finest aroma to go up the chimney. One-quart size is $1.50, and fifty cents for each additional quart. Ten-quart sizes, $4.50.

---

We believe we have the most honest set of subscribers in the United States. "Reason cause," as our little Alice says, they have not a chance to do otherwise, as the Journal is never sent without an order *and* a dollar. But more than that, by some means two numbers were sent to a subscriber for some months: "I inclose another dollar for the extra number," but that is no more than one would expect from sturdy old North Carolina. Another writes: "I do not know whether I have sent my subscription or not, if I have, the inclosed dollar will pay for the Journal for 1858, for my friend, whose address I append." *Per contra*, a gentleman from Detroit sent us, last year, *two* dollars, and we sent back one!

